# The Prognostic and Risk-Stratified Value of Heart-Type Fatty-Acid-Binding Protein in Community Acquired Pneumonia in Emergency Department

**DOI:** 10.1155/2014/753070

**Published:** 2014-07-16

**Authors:** Yun-Xia Chen, Chun-Sheng Li

**Affiliations:** Emergency Department of Beijing Chao-Yang Hospital, Affiliated to Capital Medical University, Chaoyang District, Beijing 100020, China

## Abstract

*Objective.* To evaluate the prognostic and risk stratified ability of heart-type fatty-acid-binding protein (H-FABP) in patients with community acquired pneumonia (CAP) in emergency department (ED) and to compare it with Pneumonia Severity Index (PSI) and CURB-65.* Methods.* Consecutive adult CAP patients admitted to the ED of Beijing Chao-Yang Hospital were enrolled. Circulating H-FABP and troponin I were measured. PSI and CURB-65 were calculated in all patients. The differences in 28-day mortality and requirement for mechanical ventilation (MV) or a vasopressor within 6 h after ED arrival were compared in patients with positive H-FABP (≥7 ng/mL) and negative ones (<7 ng/mL). Receiver operating characteristic (ROC) curve and logistic regression were used to assess the predictive value of H-FABP.* Results.* From August to November 2012, 229 CAP patients were enrolled. The 28-day mortality, PSI, CURB-65, and incidence of using MV or a vasopressor were much higher in H-FABP-positive patients than in negative ones (*P* < 0.01). H-FABP was an independent predictor of the 28-day mortality. The area under the ROC curve (AUC) of H-FABP was 0.751. Combination of H-FABP and CURB-65 (AUC = 0.824) or H-FABP and PSI (AUC = 0.820) improved their prognostic performance.* Conclusions.* H-FABP was valuable for prognosis and risk stratification in CAP patients in ED.

## 1. Introduction

Community acquired pneumonia (CAP) is a common reason of seeking emergency care and ICU admission. It is a major healthcare problem, affecting millions of people in different countries each year and increasing morbidity and mortality [1–4]. In the management of CAP, assessment of disease severity and prediction of outcome are essential for a rational allocation of health care resources and for guiding therapeutic options. For this purpose, different risk assessment tools have been developed. The Pneumonia Severity Index (PSI) and CURB-65 are most widely used risk assessment tools [5, 6], and both of them are valuable in prognosis and risk stratification of CAP. However, they have some limitations. PSI is complex to calculate, overemphasizes age and comorbidities, and excludes risk factors such as COPD and diabetes. CURB-65 underestimates severity in young patients and does not take into account comorbidities. Both of them perform less well for need for ICU/ventilatory or vasopressor support [7]. Recent studies demonstrated that combination of biomarker and severity score system improved the accuracy in predicting outcomes [8, 9].

Heart-type fatty-acid-binding protein (H-FABP) is a sensitive and specific biomarker of myocardial injury and superior to traditional biomarkers for the assessment of recurrent or persistent myocardial damage [10–12]. Recent study revealed that H-FABP independently predicts 28-day mortality in severe sepsis [13]. The prognostic and risk-stratified value of H-FABP in CAP was not reported yet.

The present study was designed to evaluate the prognostic and risk stratified ability of H-FABP in patients with CAP in emergency department (ED) and to compare it with PSI and CURB-65.

## 2. Methods

### 2.1. Patients

From August to November 2012, consecutive patients admitted to the ED of Beijing Chao-Yang Hospital were enrolled. The enrollment criteria were as follows: age > 18 years and fulfillment of the criteria for CAP defined in Guidelines [14]. The exclusion criteria included age < 18 years; myocardial injury induced by other diseases except CAP (such as acute coronary syndrome [ACS], myocarditis, defibrillation, direct current cardioversion, chest compression, thorax trauma, thoracotomy, cardiac or hemorrhagic shock, and acute or chronic heart failure); chronic renal dysfunction; muscle disorders; and patients or their relatives who declined to participate in the study. Patients suffering from ischemic chest pain and with elevated troponin level were excluded and took further examinations for ACS. Patients with Wells Clinical Prediction score of 2 or more were suspected to be suffering from pulmonary embolism (PE) and excluded [15].

The present study was approved by the hospital Ethics Committee. Written informed consent was obtained from all patients.

### 2.2. Data Collection

The comorbidities and vital signs of patients were recorded at enrollment. Laboratory examinations including full blood count, serum chemistry, blood gas analyses, and troponin I (TNI) were examined and recorded on ED arrival. The PSI and CURB-65 were calculated at enrollment.

### 2.3. Measurement of H-FABP

H-FABP was measured using a colloidal gold rapid test strip of human H-FABP (Kang Sheng Bao Bio-Technology Co., Ltd., Shenzhen, China). Blood samples were collected from the median cubital vein of patients and three drops (~120 *μ*L) of whole blood was added to the test strip. The result was shown as the appearance of one or two red bands in the test card window after 15 minutes. Two red bands at the test and control zones were a positive result; one band at the control zone was a negative one. No band develops at both zones if the test was invalid. The test strip was then placed into the QuickSens Omega 100 analyzer (8sens biognostic GmbH, Berlin, Germany), and the level of H-FABP could be read on the screen.

### 2.4. Outcome Variables

The enrollment patients were followed up for 28 days by reviewing medical records or telephone. The primary outcome was 28-day mortality. Secondary outcome variables included requirement for mechanical ventilation (MV) or vasopressor within 6 h after ED arrival.

### 2.5. Statistical Analysis

Data were analyzed using SPSS version 13.0 (SPSS Inc., Chicago, IL, USA). Normal distribution data were expressed as mean ± standard deviation and analyzed using the Student's* t*-test. Skewed distribution data were expressed as median and quartiles and compared using the Mann-Whitney *U* test. Categories were assessed with the chi-square test for independence. Logistic regression analysis was adopted in determining the independent predictors of outcomes. The predictive values of independent predictors were tested using the receiver operating characteristic (ROC) curve and area under the curve (AUC). The cutoff values were determined by ROC curve, and sensitivity, specificity, positive predictive value (PPV), negative predictive value (NPV), and positive (LR+) and negative (LR–) likelihood ratios were also calculated. A Kaplan-Meier curve was used to illustrate the cumulative proportions of survival, and the difference between groups was tested using the log-rank test. A two-tailed *P* < 0.05 was considered statistically significant.

## 3. Results

### 3.1. Patient Characteristics

At the period of enrollment, 284 patients were assessed and 45 were excluded. By the end of the 28-day follow-up, 10 patients were missed, and 229 patients were enrolled. The enrolled patients were separated into H-FABP-positive group (≥7 ng/mL) or H-FABP-negative group (<7 ng/mL). As seen in Table 1, there was no difference in age and gender between the two groups.

PSI and CURB-65 scores were higher in H-FABP-positive patients. The 28-day mortality and the incidence of using MV or a vasopressor within 6 h after ED arrival were higher in H-FABP-positive patients, too. The survival time of H-FABP-positive patients was shorter than H-FABP-negative ones.

### 3.2. H-FABP Levels in Patients with Different Outcome

**Figure 1:**
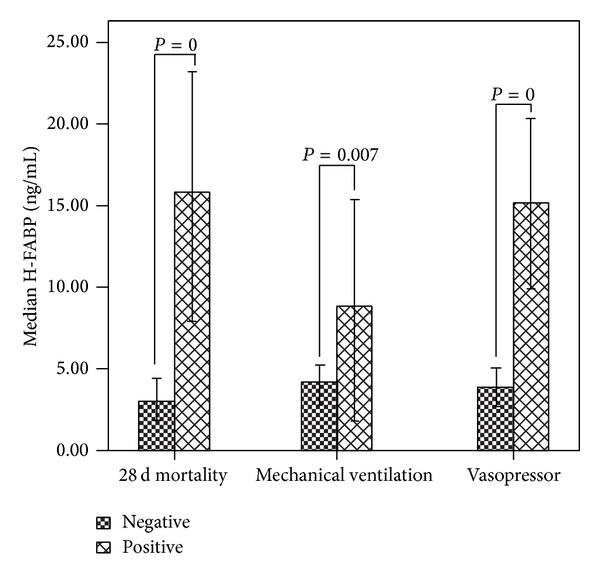
Heart-type fatty-acid-binding protein (H-FABP) levels in patients with different outcomes. Circulating H-FABP level was higher in patients who died within 28 days (positive) after emergency department (ED) arrival than in patients who did not (negative) (*P* = 0). It was also higher in patients who required mechanical ventilation (*P* = 0.007) or a vasopressor (*P* = 0) within 6 h after ED arrival (positive) than in those who did not (negative).

As shown in Figure 1, patients who needed MV within 6 h after ED arrival had a higher H-FABP level compared with those who did not (8.845 (1.806–15.525) versus 4.163 (0.985–9.787), *P* = 0.007). H-FABP levels in patients who required a vasopressor within 6 h after ED arrival were much higher than in those who did not (15.160 (6.236–22.090) versus 3.845 (0.955–8.569), *P* = 0). H-FABP levels were much higher in nonsurvivors than in survivors (15.810 (4.077–31.230) versus 2.956 (0.770–7.636), *P* = 0).

### 3.3. Independent Predictors of Outcomes

The independent predictors of outcomes were listed in Table 2. The independent predictors of 28-day mortality included H-FABP, PSI, and CURB-65. The independent predictors of requirement MV were age and PSI. The only independent predictor of using a vasopressor was CURB-65.

### 3.4. Prognostic and Risk-Stratified Value of H-FABP

**Figure 2:**
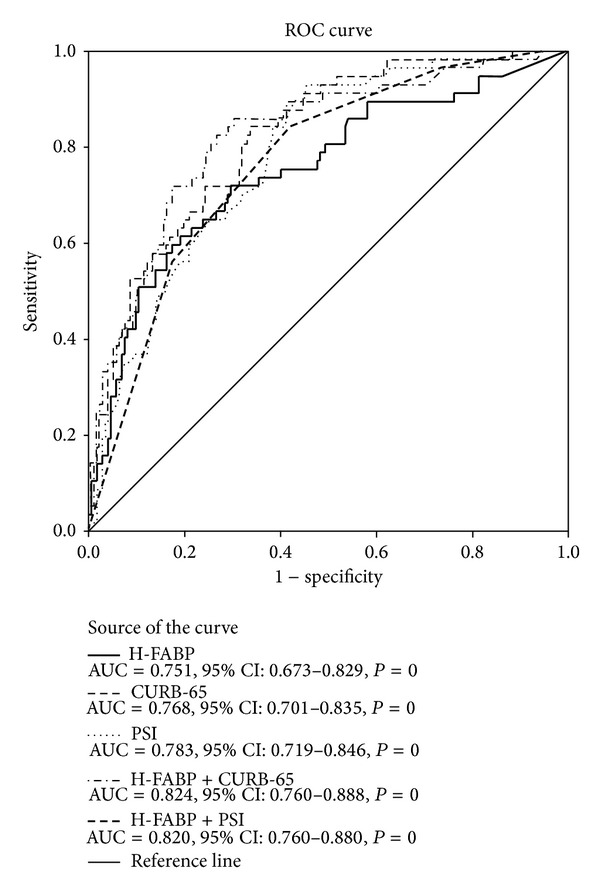
Receiver operating characteristic (ROC) curves for predicting 28-day mortality. Areas under the ROC curves (AUCs) are shown along with 95% confidence intervals (CI) and *P*  values. The differences in AUC between predictors were not significant.

The ROC curves of H-FABP, PSI, and CURB-65 predicting 28-day mortality were shown in Figure 2. The ROC curves of combination H-FABP and PSI, H-FABP, and CURB-65 were also shown in Figure 2. Combination of H-FABP and PSI (H-FABP+PSI) or H-FABP and CURB-65 (H-FABP+CURB-65) increased the accuracy in predicting risk of 28-day mortality (AUC = 0.820 and 0.824, resp.). Both of the AUCs of H-FABP+PSI and H-FABP+CURB-65 were higher than that of H-FABP, PSI, or CURB-65 alone, but the differences of AUCs between the predictors were not significant.

The sensitivity, specificity, PPV, NPV, LR+, and LR− were listed in Table 3. The cutoff value of H-FABP defined by ROC curve was 6.138 ng/mL, which was below the positive threshold (7 ng/mL) recommended by the reagent. The predictive value of the positive threshold (7 ng/mL) was assessed, and the result demonstrated that sensitivity, specificity, PPV, NPV, LR+, and LR− of it were similar to those of the cutoff value defined by ROC curve. The 28-day mortality in patients with positive H-FABP was fivefold higher than in those with negative H-FABP.

**Figure 3:**
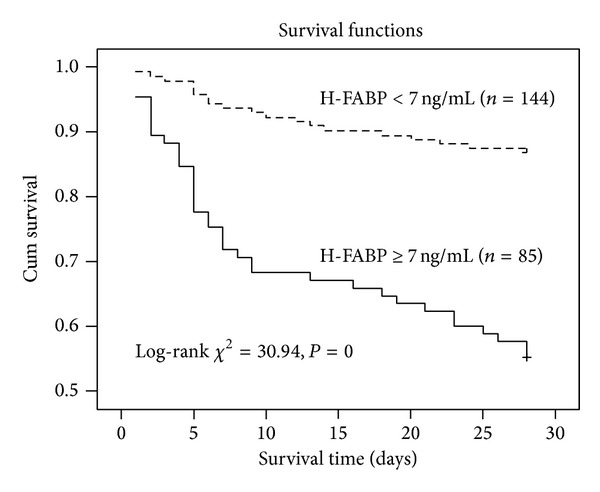
Kaplan-Meier curves of heart-type fatty-acid-binding protein (H-FABP). The survival rate of patients with positive H-FABP was higher than that of patients with negative H-FABP (*χ* ^2^ = 30.94, *P* = 0).

The Kaplan-Meier survival curves of H-FABP were shown in Figure 3. The mean survival time was 19.7 ± 10.9 and 25.8 ± 6.4 days in patients with positive and negative H-FABP, respectively. The difference in survival time between the two groups was significant (*P* = 0). The survival rate in patients whose H-FABP was above 7 ng/mL was lower than in those whose H-FABP was normal (55.3% versus 86.8%, *P* = 0).

## 4. Discussion

Respiratory infection composes the main cause of ED visit, and CAP is the most common diagnosis. The morbidity and mortality of CAP remain increasing worldwide in recent years, and CAP is still a heavy economic burden [16–19]. Early detection, appropriate risk stratification, timely intervention, proper location, and accurate outcome prediction are all crucial to improving outcome and reducing cost of CAP.

Cardiac complications were common in patients with CAP, and it was the independent predictor of short-term mortality [20]. CAP may induce vascular endothelium injury, myocardium depression, acute cardiac arrhythmia, acute inflammatory changes in atherosclerotic plaques, and coronary vasoconstriction; all these changes may result in circulating dysfunction and increase in mortality [21]. Myocardial biomarkers increase during course of disease and correlate with the severity of CAP. As ACS and PE were also commonly encountered in ED, the present study excluded patient suffering from ischemic chest pain with elevated troponin level and patients whose Wells Clinical Prediction score was 2 or more [15].

H-FABP is a novel biomarker of myocardial injury. It appears in the blood as early as 1.5 h after onset of symptoms of myocardial injury, peaks around 6 h, and returns to baseline values in 24 h [22]. These features of H-FABP make it superior to traditional biomarkers for the early detection and monitoring of myocardial damage [11]. So it is worthy to investigate the prognostic and risk-stratified value of H-FABP in patients with CAP.

The results of the present study revealed that the severity of myocardial injury (indicated by TNI), severity of illness (indicated by PSI and CURB-65), and incidence of deterioration (indicated by the requirement of MV or a vasopressor) were much higher in patients with positive H-FABP than in those with negative one. The 28-day mortality of H-FABP-positive patient was much higher than H-FABP-negative ones, and the survival time of the former was much shorter than the latter; both of the results indicated that positive H-FABP was a predictor of adverse outcome.

Quantitative measurement of H-FABP revealed that H-FABP was much higher in patients who died within 28 days and needed MV or a vasopressor within 6 h after ED arrival than in those who did not. These results demonstrated that high level H-FABP indicated more severe illness, higher tendency to deterioration, and higher risk of death from another aspect.

The present study found that H-FABP was the independent predictor of 28-day mortality, but TNI was not. The AUC of H-FABP predicting mortality in our study (0.751) was near to the result (AUC = 0.805) of a research conducted in severe sepsis. In the latter study, TNI as a consecutive variable was not the independent predictor of 28-day mortality either [13]. The prognostic ability of H-FABP was similar to PSI and CURB-65 in our study. Combination of H-FABP and PSI and combination of H-FABP and CURB-65 both improved the accuracy in predicting 28-day mortality. These results proved the possibility of adding H-FABP to a severity score system in the further researches.

Although H-FABP was much higher in patients who required MV or a vasopressor within 6 h after ED arrival than in patients who did not, it was not the independent predictor of the two outcomes. PSI and CURB-65 were more predictive than H-FABP in these situations. The main cause may be that H-FABP less correlated with acute respiration or circulation dysfunction.

## 5. Limitations

The present study was a single-center study and contained a small sample size. The results should be verified by well-designed, larger, multicenter clinical studies.

## 6. Conclusions

H-FABP independently predicts 28-day mortality in patients with CAP in ED. Positive H-FABP indicates more severe disease, higher tendency to deterioration, higher risk of death, and greater probability of using MV or vasopressors. H-FABP is valuable for prognosis and risk stratification of CAP in ED.

## Figures and Tables

**Table 1:** Patients characteristics.

	H-FABP ≥ 7 ng/mL	H-FABP < 7 ng/mL	*P*
*n*	85	144	
Age (years)	77 (61–81)	73 (61–79)	0.092
Male	47 (55.3%)	85 (59.0%)	0.581
TNI (ng/mL)	0.12 (0.05–0.71)	0.03 (0.00–0.10)	0
CURB-65	3 (2–4)	2 (1–3)	0
PSI	138.5 ± 37.5	114.3 ± 38.0	0
MV	15 (17.6%)	9 (6.3%)	0.007
Vasopressor	19 (22.4%)	6 (4.2%)	0
28-day mortality	38 (44.7%)	19 (13.2%)	0
Survival time (days)	19.7 ± 10.9	25.8 ± 6.4	0

H-FABP: heart-type fatty-acid-binding protein; TNI: troponin I; PSI: Pneumonia Severity Index; MV: mechanical ventilation.

**Table 2:** The independent predictors of outcomes.

Outcomes	Predictors	*B*	S.E.	Wald	*P*	Exp(*B*)	95% CI	
							5%	95%
28-day mortality	H-FABP	0.038	0.011	12.260	0.000	1.038	1.017	1.061
	PSI	0.016	0.007	5.171	0.023	1.016	1.002	1.030
	CURB-65	0.546	0.231	5.571	0.018	1.727	1.097	2.718
	Constant	−4.365	1.092	15.983	0.000	0.013		

Mechanical ventilation	Age	−0.046	0.017	6.909	0.009	0.955	0.923	0.988
	PSI	0.017	0.008	3.987	0.046	1.017	1.000	1.034
	Constant	−2.389	1.123	4.524	0.033	0.092		

Using vasopressor	CURB-65	1.037	0.328	10.022	0.002	2.821	1.484	5.360
	Constant	−4.541	1.404	10.455	0.001	0.011		

SE: standard error; CI: confidence interval; H-FABP: heart-type fatty-acid-binding protein; PSI: Pneumonia Severity Index.

**Table 3:** Cutoff values of H-FABP.

Cutoff value	Sensitivity	Specificity	PPV	NPV	LR+	LR−	OR	95% CI	
(ng/mL)								5%	95%
6.138	71.9%	70.3%	44.6%	88.3%	2.43	0.4	6.080	3.130	11.810
7	66.7%	72.7%	44.7%	86.8%	2.44	0.46	5.319	2.791	10.136

H-FABP: heart-type fatty-acid-binding protein; PPV: positive predictive value; NPV: negative predictive value; LR+: positive likelihood ratio; LR−: negative likelihood ratio; OR: odd ratio; CI: confidence interval.
